# Coexisting giant splenic artery and portal vein aneurysms leading to non-cirrhotic portal hypertension: a case report

**DOI:** 10.1186/s13256-016-1059-4

**Published:** 2016-09-29

**Authors:** Abidullah Khan, Maimoona Ayub, Iqbal Haider, Mohammad Humayun, Zakir Shah, Fahad Ajmal

**Affiliations:** Resident Internal Medicine, Khyber Teaching Hospital, 25000 Peshawar, Pakistan

**Keywords:** Splenic artery aneurysm, Portal vein aneurysm, Non-cirrhotic, Portal hypertension, Hypersplenism

## Abstract

**Background:**

Splenic artery aneurysms are the commonest visceral and third most common abdominal artery aneurysms, having a strong association with both pregnancy and multiparity. Here we report possibly the first case of a giant splenic artery aneurysm in association with a smaller portal vein aneurysm, in a woman who had never conceived, leading to non-cirrhotic portal hypertension.

**Case presentation:**

A 40-year-old Pakistani Asian woman who had no evidence of liver cirrhosis presented in April 2016 for a diagnostic workup of ascites, massive splenomegaly, and pancytopenia. An abdominal ultrasound followed by computed tomography angiography showed a giant aneurysm in her splenic artery and another smaller one in her portal vein.

She underwent splenectomy and excision of the splenic artery aneurysm. Surgical findings included a giant splenic artery aneurysm pressing on her portal vein and causing its aneurysmal dilatation. On her first review in July 2016, she was generally in good health, ascites had subsided, and her full blood count was normal. Her portal vein aneurysmal dilatation, which was presumed to be secondary to the pressure effect from the splenic artery aneurysm, had shrunken remarkably in size.

**Conclusion:**

A giant splenic artery aneurysm can cause non-cirrhotic portal hypertension and should be treated with splenectomy and aneurysmectomy.

## Background

Splenic artery aneurysms (SAAs) are the commonest of all visceral aneurysms accounting for approximately 60 % of the cases [1, 2]. They are more common in women of child bearing age and usually present during pregnancy; the female to male ratio is 4:1 [3]. They can be diagnosed incidentally on abdominal imaging or more commonly present with shock and severe abdominal pain when they rupture and usually prove to be fatal [4, 5]. Non-cirrhotic portal hypertension secondary to SAA has very rarely been described in the medical literature and this case report is one such case.

We thoroughly searched for relevant literature on PubMed, Medscape, EMBASE and Web of Science by using the keywords, “splenic artery aneurysm,” “portal vein aneurysm,” and “non-cirrhotic portal hypertension.” Our literature search of electronic medical databases revealed only 400 descriptions of cases of SAAs in the English language medical literature and, interestingly, only 13 of the SAAs measured more than 10 cm in size [6, 7]. It must also be noted that to date, only 50 cases of isolated portal vein aneurysms have been reported since their first description in 1956 by Barzilai and Kleckner [8]. Our case is unusual in that there were coexisting giant splenic artery and portal vein aneurysms, measuring 12.2×9.4 cm and 3.4×2.6 cm respectively, leading to non-cirrhotic portal hypertension, ascites, and massive splenomegaly with hypersplenism in a 40-year-old non-pregnant woman, probably the first case report of its type described in the medical literature.

## Case presentation

A 40-year-old Pakistani Asian woman was admitted to the medical unit of Khyber Teaching Hospital, Peshawar, Pakistan in April 2016 for the diagnostic workup of ascites and massive splenomegaly with pancytopenia. Her retrospective history revealed that she had an episode of hematemesis 10 years previously, for which she had an upper gastrointestinal (GI) endoscopy. The endoscopy showed esophageal varices. However, after her endoscopy, she lost contact with the hospital and was not seen again. During that time interval, she admitted to having on/off upper abdominal pain.

During her current visit to our hospital, she reported melena, weight loss, mild-moderate epigastric pain, anorexia, and worsening ascites. She denied any bleeding from any other orifice, menstrual irregularities, altered bowel habits, and chest or urinary symptoms. Her family history was significant for pulmonary tuberculosis. However, she denied any fever or cough.

On examination, her vital signs were within normal limits. She was lean looking, anemic, and mildly icteric. She did not have spider nevus, palmar erythema, leukonychia, palpable lymph nodes, purpura, or any bony tenderness. However, she had tense ascites and massive splenomegaly but her liver span was normal. The rest of her general physical and systemic examination was unremarkable.

Her full blood count revealed: hemoglobin (Hb) 9.5 gm/dl, white blood cell (WBC) 4000/mm^3^, platelets 70,000/mm^3^ and reticulocyte count of 2.4 %. Her bone marrow examination was consistent with features of hypersplenism/peripheral destruction and reduced iron stores. An ultrasound of her abdomen and pelvis was notable for ascites, enlarged spleen measuring 22 cm with multiple splenic infarcts, dilated portal vein, and a large aneurysm in her splenic artery. Her liver parenchyma and its margins, and her hepatic veins, kidneys, gall bladder, pancreas, and pelvic organs were all reported to be normal. An ascitic fluid analysis revealed hemorrhagic and exudative fluid with negative polymerase chain reaction (PCR) for *Mycobacterium tuberculosis*. An ultrasound elastography (FibroScan) of her liver showed early liver fibrosis; however, her liver function tests were normal and her autoimmune profile for liver diseases such as primary biliary cirrhosis, primary sclerosing cholangitis, and autoimmune hepatitis was negative. She did not have any serological evidence of chronic hepatitis B or C. Other investigations such as erythrocyte sedimentation rate (ESR), anti-nuclear factor (ANF), Coombs test, lactate dehydrogenase (LDH), blood sugar, lipid profile, and electrolytes were normal.

A repeat upper GI endoscopy was notable for three columns of large esophageal and a bunch of fundal varices, which were band ligated under endoscopic guidance. Both computed tomography (CT) of her abdomen and pelvis followed by CT angiography of her abdominal vessels showed a giant saccular splenic artery mid segment aneurysm measuring 12.2×9.4 cm with peripheral mural thrombosis and calcification, a smaller splenic artery distal segment aneurysm, and gross splenomegaly with infarcts. Other findings included another aneurysm in her portal vein at the level of bifurcation in the porta hepatis measuring 3.4×2.6 cm and two small hepatic hemangiomas in the hepatic segments eight and five measuring 1.2 cm and 1.6 cm respectively. She was thus diagnosed as having a giant splenic artery aneurysm and a smaller portal vein aneurysm leading to non-cirrhotic portal hypertension, splenomegaly, and hypersplenism.

**Fig. 1:**
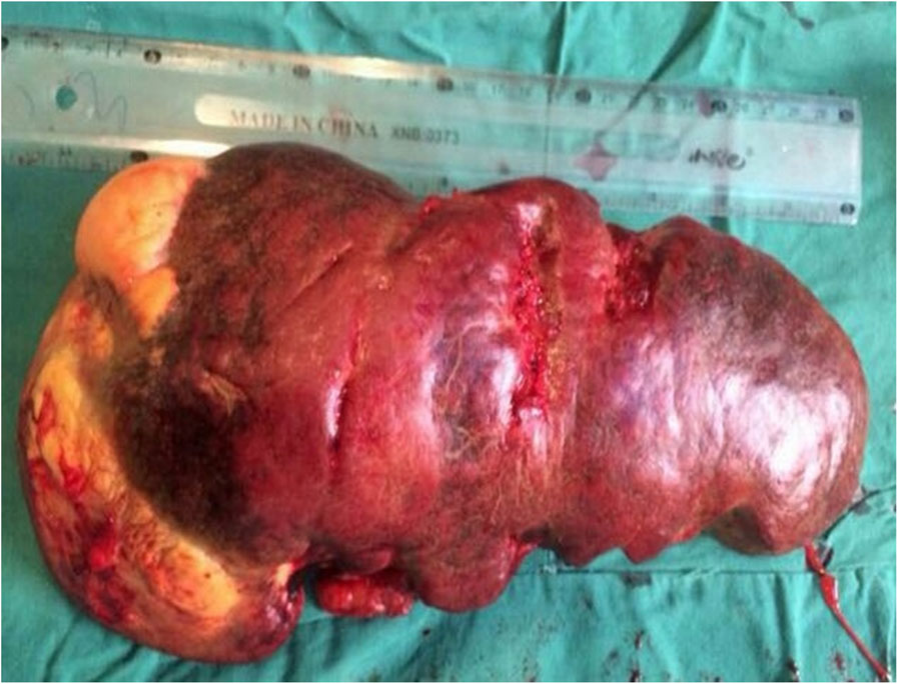
Gross view of the explanted spleen and part of the aneurysm (shown on the *left*). The photograph was taken with permission from the patient

**Fig. 2:**
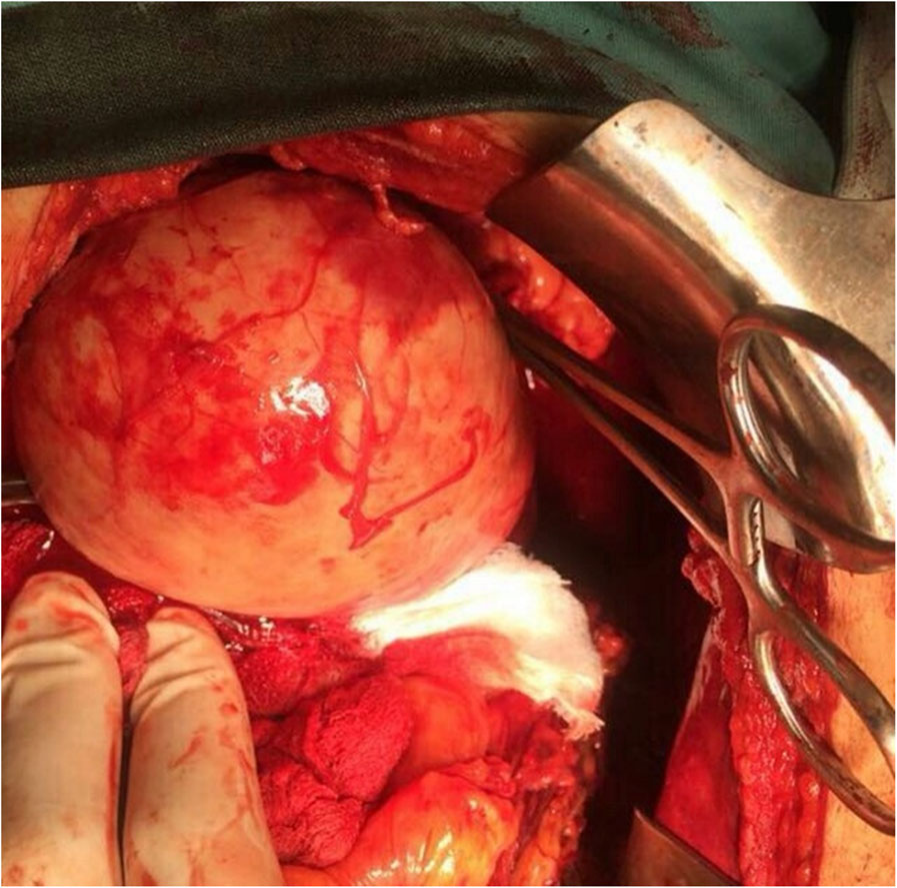
Intraoperative findings showing a giant splenic artery aneurysm which was excised. The photograph was taken with permission from the patient

She was taken to our vascular team and had a laparotomy. Elective aneurysm resection with concomitant splenectomy was performed using an upper median laparotomy and direct approach through the bursa omentalis (Fig. 1). The operative findings included a giant SAA in the mid-distal segments with a lot of adhesions in the surrounding areas, omental thickening, and a pressure effect on her portal and splenic veins leading to extrahepatic portal vein obstruction and resultant portal hypertension. The SAA, which was heavily calcified and embedded in her pancreatic tissue, had evidence of intramural hematoma and perianeurysmal blood leakage (Fig. 2). The size of the aneurysm and active bleeding from the aneurysmal sac distorted and obscured local anatomy, precluding selective ligation of her proximal splenic artery and making supraceliac aortic clamping before opening of the aneurysmal sac necessary.

Her portal vein aneurysmal dilatation was assumed to be secondary to the pressure effect caused by the SAA and was left unattended. During the operation she developed shock due to massive intraoperative bleeding and received approximately 3410 mL (6 pints) of packed cells and inotropic support. She was moved to our surgical Intensive Care Unit (ICU) where she had an uneventful recovery. Histopathology of the excised SAA revealed signs of medial necrosis.

She was reviewed 2 months after the operation. On her first follow-up visit in July 2016, she was generally in good health. She reported symptomatic improvement. Her ascites had subsided and her full blood count had returned to normal. A repeat CT angiography showed a remarkable reduction in the size of portal vein aneurysmal dilatation. Her portal vein aneurysm had regressed remarkably, from a size of 3.4×2.6 cm before splenectomy to a size of 1.3×1.1 cm after splenectomy. We will review her again after 6 months. Duplex ultrasound and/or CT angiography will be used to monitor her in general and her portal vein aneurysmal dilatation in particular.

## Discussion

**Fig. 3:**
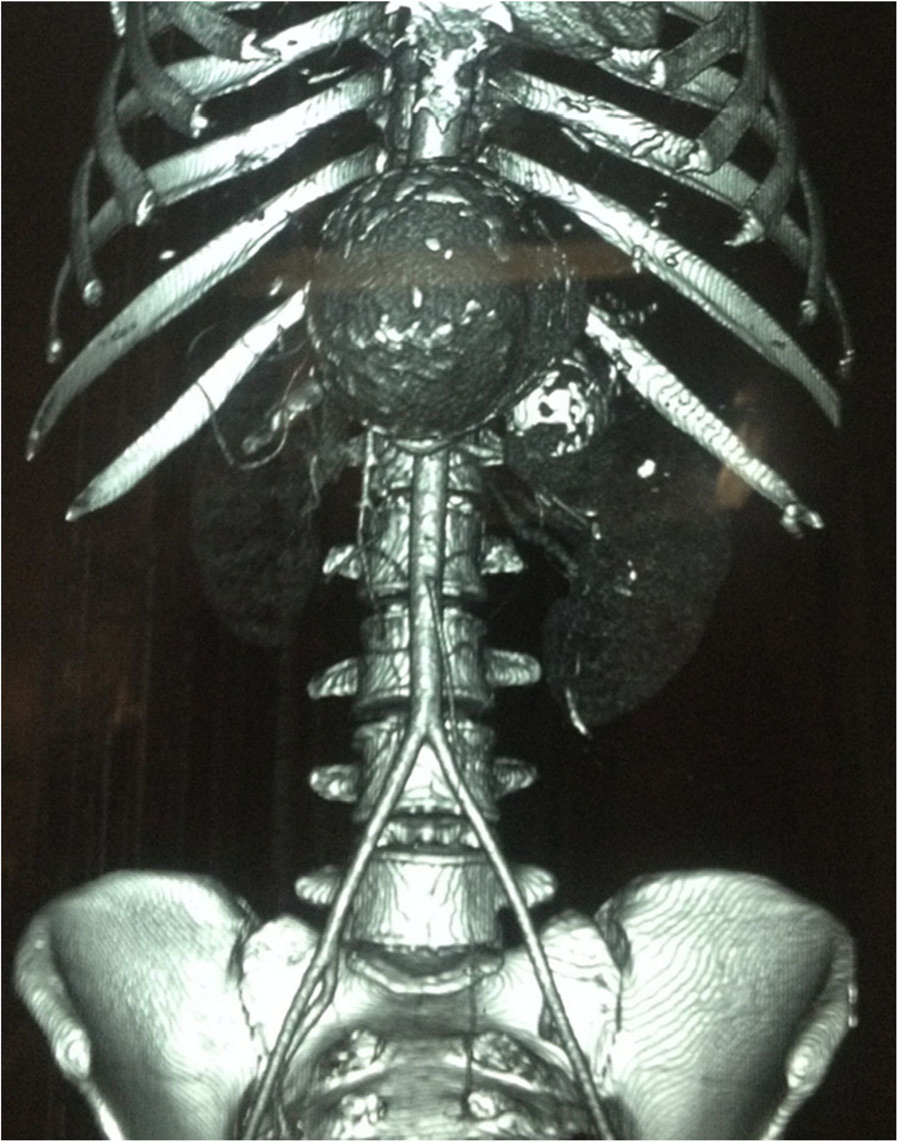
Computed tomography angiogram of our patient showing a giant splenic artery aneurysm. The computed tomography angiogram was taken with permission from our patient

The reported mean age of incidence of SAAs is 52 years and annual incidence ranges from 0.01 to 0.2 % [9]. Most of the SAAs were small, measuring less than 2 cm, arose at the site of arterial bifurcation, and were frequently found at a mid or distal segment of the splenic artery. SAAs greater than 2.5 cm have been termed giant SAAs and those measuring 10 cm or more are even rarer [1, 7, 10]. In our case the SAA was huge measuring 12.2×9.4 cm and it was located at the middle segment of the splenic artery with peripheral mural calcification and thrombosis (Fig. 3).

The pathophysiology of SAAs is largely unknown. There has been a proposed association of SAA with pregnancy. It is postulated that high levels of estrogen and progesterone along with increased cardiac output and high volume status during pregnancy lead to alteration in the arterial wall and defective elastin synthesis, which makes the splenic artery more likely to dilate. Other potential risk factors include portal hypertension, vascular and connective tissue disorders, congenital abnormalities of the vessels, vascular trauma, inflammatory processes, and degenerative arterial disease. It is worth noting that our patient had never conceived and had none of the other risk factors mentioned [6].

SAAs are usually incidental findings on abdominal imaging done for another medical reason. However, they can present acutely as an emergency when they rupture leading to hypovolemic shock with very high mortality rates or insidiously in the form of non-cirrhotic portal hypertension presenting as chronic abdominal pain, splenomegaly, ascites, or hematemesis and so forth [11, 12].

SAA can be diagnosed on the basis of ultrasound with pulsed Doppler. However, arteriography or CT angiography has the highest yield and can precisely demonstrate the anatomy of the aneurysms [6]. In our case, the CT angiography showed a massive SAA which was leaking intermittently with mural calcification and thrombosis. Her portal vein was also tortuous with a small aneurysm without thrombus formation at the level of bifurcation. As portal hypertension is the most common cause for portal vein aneurysmal dilatation, we assumed that the portal vein aneurysmal dilatation in our case was secondary to non-cirrhotic portal hypertension and as a result of the pressure effect caused by the large SAA. Our assumption was also supported by the fact that the size of the portal vein aneurysm had diminished remarkably when she was reviewed in July 2016, which was just 2 months after her splenectomy and aneurysmectomy.

Treatment of SAA can be challenging and there is limited guidance regarding when and how to treat patients with SAA. This is most probably due to a lack of randomized trials as the disease itself is extremely rare. However, from the limited retrospective clinical series, it is evident that SAA needs to be treated if it is diagnosed in a pregnant woman or measures 2 cm or more, as these factors increase the risk of spontaneous rupture. Similarly, those who are liver transplant candidates must undertake treatment for SAA. Treatment of SAA includes aneurysmectomy with or without splenectomy and novel non-operative endovascular techniques. Moreover, those with non-cirrhotic portal hypertension, hypersplenism, and/or acute rupture may benefit the most from splenectomy as well as aneurysmectomy [8, 11, 13–15].

The surgical approach for giant SAAs is usually challenging because the bigger size of the aneurysm makes even the celiac trunk inaccessible anteriorly [16]. The huge size of the aneurysm in our case necessitated proximal bleeding control through clamping of the aorta at the level of the diaphragm. A thoracoabdominal incision was used by Long *et al*. to gain retroperitoneal access to the subdiaphragmatic aorta and the celiac trunk [17]. Moreover, Pescarus *et al*. recommended the retroperitoneal approach with a chevron incision and medial visceral rotation for similar reasons [18]. It is therefore recommended that while operating on patients with SAAs generally and giant SAAs specifically, careful consideration should be given to the risks and benefits of the surgical procedure. Moreover, to minimize the risk of serious complications during the operation, the type of surgical approach should be tailored according to the size and location of the lesion and be decided on a case-by-case basis.

## Conclusions

SAAs should be considered in the differential diagnoses of non-cirrhotic portal hypertension. Most of the larger SAAs have a natural tendency of spontaneous rupture which can be catastrophic. The treatment of choice is surgical and it must be considered as early as possible, especially in those with giant aneurysms, hypersplenism, or in liver transplant candidates. Splenectomy with excision of the aneurysm is the usual surgical approach with a high cure rate.

## Abbreviations

ANF: Anti-nuclear factor
CT: Computed tomography
ESR: Erythrocyte sedimentation rate
GI: Gastrointestinal
Hb: Hemoglobin
ICU: Intensive care unit
LDH: Lactate dehydrogenase
PCR: Polymerase chain reaction
SAA: Splenic artery aneurysm
WBC: White blood cell
